# Expression of Concern: Concordantly fabricated heterojunction ZnO–TiO_2_ nanocomposite electrodes *via* a co-precipitation method for efficient stable quasi-solid-state dye-sensitized solar cells

**DOI:** 10.1039/d3ra90108d

**Published:** 2023-11-09

**Authors:** Ahmed Esmail Shalan, Ahmed Mourtada Elseman, Mahmoud Rasly, Marwa M. Moharam, Monica Lira-Cantu, Mohamed M. Rashad

**Affiliations:** a Electronic and Magnetic Laboratory, Advanced Materials Division, Central Metallurgical Research & Development Institute (CMRDI) P.O. Box 87 Helwan Cairo Egypt rashad133@yahoo.com +202-25010639 +202-25010640-43; b Centro de Investigació en Nanociència i Nanotecnologia, CIN2 (CSIC-ICN), Campus UAB Edifici ETSE 2nd Floor Bellaterra Barcelona E-08193 Spain

## Abstract

Expression of concern for ‘Concordantly fabricated heterojunction ZnO–TiO_2_ nanocomposite electrodes *via* a co-precipitation method for efficient stable quasi-solid-state dye-sensitized solar cells’ by Ahmed Esmail Shalan *et al.*, *RSC Adv.*, 2015, **5**, 103095–103104, DOI: 10.1039/C5RA21822E.

The Royal Society of Chemistry is publishing this expression of concern in order to alert readers that concerns have been raised regarding the reliability of the XPS data in Fig. 5, the IPCE data in Fig. 6 (right) and the Nyquist data in Fig. 7. An investigation is underway, and an expression of concern will continue to be associated with the article until a final outcome is reached.

Laura Fisher

02/11/2023

Executive Editor, *RSC Advances*

## Supplementary Material

